# Vorolanib, sunitinib, and axitinib: A comparative study of vascular endothelial growth factor receptor inhibitors and their anti-angiogenic effects

**DOI:** 10.1371/journal.pone.0304782

**Published:** 2024-06-04

**Authors:** Sophie J. Bakri, Jeff Lynch, Michelle Howard-Sparks, Stephan Saint-Juste, Said Saim

**Affiliations:** 1 Department of Ophthalmology, Mayo Clinic, Rochester, Minnesota, United States of America; 2 EyePoint Pharmaceuticals, Inc., Watertown, Massachusetts, United States of America; 3 Department of Polymer Science and Engineering, University of Massachusetts Amherst, Amherst, Massachusetts, United States of America; University of South Carolina, UNITED STATES

## Abstract

**Purpose:**

Pathological angiogenesis and vascular instability are observed in diabetic retinopathy (DR), diabetic macular edema (DME), and wet age-related macular degeneration (wAMD). Many receptor tyrosine kinases (RTKs) including vascular endothelial growth factor receptors (VEGFRs) contribute to angiogenesis, whereas the RTK TIE2 is important for vascular stability. Pan-VEGFR tyrosine kinase inhibitors (TKIs) such as vorolanib, sunitinib, and axitinib are of therapeutic interest over current antibody treatments that target only one or two ligands. This study compared the anti-angiogenic potential of these TKIs.

**Methods:**

A kinase HotSpot™ assay was conducted to identify TKIs inhibiting RTKs associated with angiogenesis and vascular stability. Half-maximal inhibitory concentration (IC_50_) for VEGFRs and TIE2 was determined for each TKI. In vitro angiogenesis inhibition was investigated using a human umbilical vein endothelial cell sprouting assay, and in vivo angiogenesis was studied using the chorioallantoic membrane assay. Melanin binding was assessed using a melanin-binding assay. Computer modeling was conducted to understand the TIE2-axitinib complex as well as interactions between vorolanib and VEGFRs.

**Results:**

Vorolanib, sunitinib, and axitinib inhibited RTKs of interest in angiogenesis and exhibited pan-VEGFR inhibition. HotSpot™ assay and TIE2 IC_50_ values showed that only axitinib potently inhibited TIE2 (up to 89%). All three TKIs effectively inhibited angiogenesis in vitro. In vivo, TKIs were more effective at inhibiting VEGF-induced angiogenesis than the anti-VEGF antibody bevacizumab. Of the three TKIs, only sunitinib bound melanin. TKIs differ in their classification and binding to VEGFRs, which is important because type II inhibitors have greater selectivity than type I TKIs.

**Conclusions:**

Vorolanib, sunitinib, and axitinib exhibited pan-VEGFR inhibition and inhibited RTKs associated with pathological angiogenesis. Of the three TKIs, only axitinib potently inhibited TIE2 which is an undesired trait as TIE2 is essential for vascular stability. The findings support the use of vorolanib for therapeutic inhibition of angiogenesis observed in DR, DME, and wAMD.

## Introduction

Pathological angiogenesis is observed in many diseases, including cancer and wet age-related macular degeneration (wAMD). wAMD was a leading cause of blindness among adults aged 50 years and older in high-income countries between 1990 and 2020, thus significantly impacting quality of life [1]. A US-based study identified age-related macular degeneration (AMD) as the top predictor of visual impairment in adults with no refractive error [2]. Intravitreal anti-vascular endothelial growth factor (VEGF) injection (often monthly) is the most common primary procedure for patients with wAMD [3] and requires long-term continuous and proactive treatment [4–6]; however, patients with wAMD can lose vision in real-world settings despite receiving anti-VEGF therapy, often due to undertreatment [7]. Leading causes of undertreatment include high burden of injections and logistic burden regarding caregivers, time involved, and travel costs for frequent physician visits [8]. Disease recurrence and lack of long-term benefits result from poor treatment adherence and persistence among patients.

wAMD is a multifactorial, complex disease involving angiogenesis, inflammation, and fibrosis. Progression of wAMD occurs due to the degeneration of retinal pigment epithelium (RPE) cells in the aging retina, inadequate control of choroidal neovascularization, and fibrosis [9]. Hypoxia is common to all ischemic retinal vascular diseases, and its presence induces the synthesis of VEGF, a critical factor mediating progression to wAMD. VEGF-driven angiogenesis can lead to choroidal neovascularization and an associated increase in vascular permeability [10–12].

Multiple cell-signaling pathways participate in the development of wAMD. In addition to VEGF, the TIE2 receptor is of interest in angiogenesis, as it maintains blood vessel stability. TIE2 is influenced by two key peptide ligands: angiopoietin-1, which functions as a receptor agonist, and angiopoietin-2, which acts primarily as a receptor antagonist, but can function as an agonist in certain circumstances [13, 14]. Activation of TIE2 ensures vascular health and stability by activating signaling pathways that result in decreased vascular permeability and inflammation [13, 14].

**Fig 1:**
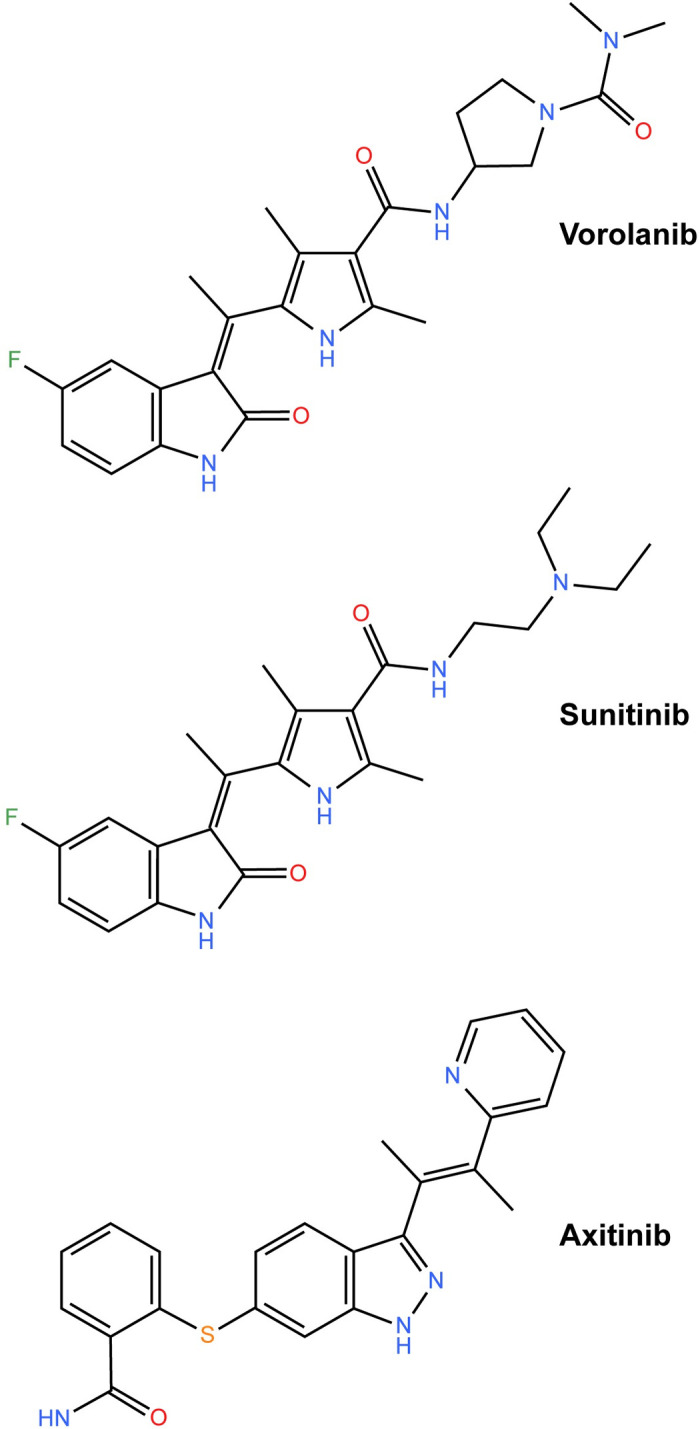
Chemical structures of the three TKIs included in this study. Structures were created using KingDraw (Qingdao, China). TKI, tyrosine kinase inhibitor.

Current antibody-based wAMD therapies only target a small number of VEGF ligands (i.e., VEGF-A and VEGF-B.) Small-molecule tyrosine kinase inhibitors (TKIs) such as vorolanib, sunitinib, and axitinib (Fig 1) offer advantages over antibody treatments for wAMD by targeting multiple VEGF receptors (VEGFRs) intracellularly [15]. Sunitinib capsules and axitinib tablets have been approved by the US Food and Drug Administration for the treatment of certain types of cancer [16, 17], and vorolanib tablets in combination with everolimus were approved in 2023 by China’s National Medical Products Administration for the treatment of advanced renal cell carcinoma [18]. Type I TKIs, including sunitinib, mimic adenosine triphosphate (ATP), only bind active receptor tyrosine kinases (RTKs), and are considered non-selective. Type II TKIs such as axitinib indirectly compete with ATP, inhibiting ATP binding by RTKs via steric hindrance. Type II TKIs bind inactive RTKs and are more selective because inactive RTK conformations display higher variability [19]. Vorolanib, a more recently developed TKI that is a multikinase inhibitor of VEGFRs and similar to axitinib and sunitinib, has shown potent anti-angiogenic activity through the targeting of multiple pathways associated with neovascular pathologies [20, 21]. Vorolanib was chemically derived from sunitinib, but it has a shorter half-life and limited tissue accumulation compared to sunitinib and other TKIs [21, 22]. Vorolanib was designed to have a more favorable safety profile, with higher selectivity and less potency against off-target kinases versus sunitinib [21].

In the human eye, melanin is present in the iris, choroid, and RPEs, and melanin turnover in some of these ocular tissues is thought to be absent [23]. Melanin binding by a drug does not predict retinal toxicity [24], but tissue accumulation may be a concern if accumulated drug impacts normal cell function. Although there has been an awareness of melanin binding by drugs for decades, it remains poorly understood [25]. The effect of melanin binding on drug distribution in the back of the eye has been reported and may lead to significantly elevated drug concentrations in pigmented tissues compared to non-pigmented tissues [25]. Melanin binding affects free drug concentrations, and the impact of melanin binding on drug safety is controversial. Because the physiological impact of melanin binding by drugs is poorly understood, it is therefore a significant factor that should be considered for pharmacokinetics and drug delivery strategies.

TKI treatments are of great therapeutic interest in the fields of oncology and ophthalmology. Orally administered vorolanib has been studied in patients with wAMD [26–28]., Gastrointestinal and hepatobiliary adverse events (AEs) were reported in a phase 2 study in wAMD [27], however, no ocular AEs were reported. The literature reports AEs following systemic axitinib administration such as impaired retinal circulation [29], bilaterial retinal hemorrhage [30], and retinal vein occlusion [31]; however, the underlying mechanism to explain these AEs associated with systemic axitinib has not yet been fully elucidated. Intravitreal administration of a TKI for treatment of wAMD delivers the drug directly to the target tissue, thereby reducing the dose and substantially reducing systemic exposure and potential for systemic effect. There are several sustained-release TKIs utilizing different polymers which have been tested for clinical stage development for wAMD, and these include: EYP-1901 (vorolanib), EyePoint Pharmaceuticals; GB-102 (sunitinib), Graybug Vision; OTX-TKI (axitinib), Ocular Therapeutix; and CLS-AX (axitinib), Clearside Biomedical. When a TKI is delivered directly into the eye by intravitreal injection, clinical trials have shown a positive ocular safety profile as evidenced by: 1) a 17-patient phase 1 trial conducted by EyePoint Pharmaceuticals [32, 33]; 2) a 21-patient phase 1 trial conducted by Ocular Therapeutix [34, 35]; and 3) a 160-patient phase 2 trial conducted by EyePoint Pharmaceuticals [36]. For wAMD treatment, the ideal sustained-delivery TKI therapy would be an intravitreally administered drug with a favorable safety profile that delivers therapeutic concentrations to the ocular tissues, which could serve to maintain vision while lowering the anti-VEGF treatment burden for patients.

There are currently no approved pan-VEGFR TKIs for the treatment of ocular diseases, but there are several presently in clinical trials. This collection of studies was conducted to compare the RTK inhibition and anti-angiogenic potential of three TKIs, vorolanib, sunitinib, and axitinib, all of which are of interest for the treatment of wAMD and other VEGF-driven ocular diseases.

## Materials and methods

### Compounds

Vorolanib in the S configuration was provided by Betta Pharmaceuticals (Zhejiang, China). Sunitinib and axitinib were provided by Adooq Bioscience (Irvine, California).

### Kinase screening panel

Measurement of kinase activity was performed by Reaction Biology Corporation (Reaction Biology Corporation, Malvern, PA), using the HotSpot™ assay. The screen focused on RTKs reported in the literature to play a major role in the regulation of angiogenesis (VEGFR1, VEGFR2, and VEGFR3; fibroblast growth factor receptor [FGFR] 1, 2, and 3; and platelet-derived growth factor receptor beta [PDGFRβ]) as well as an RTK essential for maintaining blood vessel stability (TIE2). For the profiling, specific kinase/substrate pairs and required cofactors were prepared in a reaction buffer (20mM Hepes [pH 7.5], 10mM MgCl_2_, 1mM EGTA, 0.02% Brij35, 0.02 mg/mL BSA, 0.1mM Na_3_VO_4_, 2mM DTT, 1% dimethyl sulfoxide [DMSO]). TKI compounds were diluted to 10μM and 1μM concentrations for the screens and after addition to the reaction buffer incubated for 20 minutes, followed by the addition of a mixture of ATP (Sigma, St. Louis, MO) and ^33^P-ATP (Perkin Elmer, Waltham, MA) to a final ATP concentration of 10μM. Reactions were carried out at room temperature for 2 hours, followed by spotting of the reactions onto P81 ion exchange filter paper (Whatman Inc., Piscataway, NJ). Unbound phosphate was removed by extensive washing of the filters. After subtraction of the background derived from control reactions containing inactive enzyme, kinase activity data were expressed as the percent remaining kinase activity in test samples relative to vehicle (DMSO) reactions. Kinome TREEspot images representing levels of inhibition were prepared using Kinome Mapper (https://www.reactionbiology.com/resources/tools/kinase-mapper).

### IC_50_ measurements

The half-maximal inhibitory concentration (IC_50_) studies were conducted at both Millipore Corporation (Dundee, Scotland) and at Reaction Biology Corporation (www.reactionbiology.com, Malvern, PA). All TKI compounds were prepared as 10mM stock solutions and tested in duplicate in a ten-dose IC_50_ model with a three-fold serial dilution starting at 10μM. Notably, IC_50_ protocols were standard methods and not fully optimized for each individual receptor. All reactions were carried out using 10μM ATP. Curve fits and IC_50_ values were obtained using PRISM^®^ software (GraphPad).

### Human umbilical vein endothelial cell (HUVEC) sprouting assay

The three TKI compounds were tested by Reaction Biology Europe (Freiburg, Germany) in the spheroid-based cellular angiogenesis assay to determine their effect on VEGF-A–induced sprouting of HUVECs. Primary HUVECs from pooled donors at passage 3 to 4 (PromoCell, Heidelberg, Germany) were grown in endothelial cell growth and basal medium (ECGM/ECBM, PromoCell). Test compounds were prepared as 100-fold concentrated stock solutions in DMSO and pre-diluted in basal medium. HUVECs were stimulated using 25 ng/mL VEGF-165 (Reaction Biology Europe, Freiburg, Germany) pre-diluted in basal medium.

Spheroids were prepared as described [37] by pipetting 400 HUVECs in a hanging drop on plastic dishes to allow overnight spheroid aggregation. Fifty HUVEC spheroids were seeded in 0.9 mL of a collagen gel and pipetted into individual wells of a 24-well plate to allow polymerization. Test compounds and VEGF-165 were added after 30 minutes by pipetting 100 μL of a ten-fold concentrated solution onto the polymerized gel. Plates were incubated at 37°C for 24 hours, and cells were fixed by adding 4% paraformaldehyde (Roth, Karlsruhe, Germany). Sprouting intensity of HUVEC spheroids treated with the test samples was quantitated using an image analysis system to determine the cumulative sprout length (CSL) per spheroid. Images of single spheroids were captured using an inverted microscope and the digital imaging software NIS-Elements BR 3.0 (Nikon). Spheroid pictures were uploaded to Wimasis (www.wimasis.com) for image analysis. The CSL of each spheroid was determined using the imaging analysis tool WimSprout. The mean CSL of ten randomly selected spheroids was analyzed as an individual data point.

### Chick chorioallantoic membrane (CAM) assay

The gelatin sponge-CAM assay was performed by Inovotion (La Tronche, France). Fertilized White Leghorn eggs (Hubert, Guilberville, France) were incubated at 37.5°C with 50% relative humidity for 8 days. At day 8 of embryonic development the CAM was dropped down by drilling a small hole through the eggshell into the air sac, and a 1-cm² window was cut in the eggshell above the CAM. A 1-mm^3^ gelatin sponge (BloXang, Bausch + Lomb) was administered on the CAM and loaded with 100 ng (10 μL) of VEGF-165 (PHC9391, ThermoFischer). The window was sealed with tape, and eggs were returned to the incubator. At day 9 of embryonic development, eggs were randomized into groups of at least 20 per experimental condition. Gelatin sponges were treated with 0.1% DMSO diluted in phosphate-buffered saline (PBS) as a negative control, 0.5 mg/kg bevacizumab (20.15μM) as a reference compound (i.e., a positive control), or TKI (vorolanib, sunitinib, or axitinib) at a dose equal to their individual VEGFR2 IC_50_, or 200 times their VEGFR2 IC_50_. VEGFR2 IC_50_ values for vorolanib (52nM) and sunitinib (43nM) were determined by Millipore Corporation (Dundee, Scotland) prior to the study and are cited in the literature [27] while the VEGFR2 IC_50_ value for axitinib (0.2nM) is also documented in the literature [27, 28]. Egg monitoring was performed daily until day 12 of embryonic development. Sponges were then photographed in ovo with an Olympus SZX16 microscope equipped with the Olympus XC50 camera system. Blood vessels entering the sponges within the focal plane of the CAM were counted by three technicians in a triple-blinded fashion at magnification ×1.6. A one-way statistical analysis of variance (ANOVA) with post-tests was performed using GraphPad PRISM^®^ 9 software.

For scoring of vessel diameter, photographs of vessels perfusing each sponge were examined and scored by three technicians. Eight photographs per group were included in the analysis, and vessel diameter was graded using a semi-quantitative scoring system. The scoring was defined as follows based on the percentage of vessels reaching the sponges having a standard diameter: Score 0, 100%; Score 1, ≥ 75%; Score 2, ≥ 50%; Score 3, ≥ 25%. The mean score of each experimental compound was compared to the mean score of the negative control group. A one-way statistical ANOVA with post-tests was performed using GraphPad PRISM^®^ 9 software.

### Melanin-binding assay

A melanin-binding assay was completed by Charles River Laboratories (Worchester, MA). Test compounds (vorolanib, sunitinib, and axitinib) and chloroquine (positive control) were tested for binding to synthetic melanin at concentrations ranging from 0.0620 to 25.0μM. Compound stock solutions were prepared in DMSO and further diluted in PBS with and without melanin to the specified concentrations and incubated at 37°C for 1 hour. Aliquots (50 μL each) of 2× assay samples were utilized. Aliquots of PBS without melanin samples were quenched at time zero to be used as stability controls and calibration standards. Free compound was separated from melanin-bound compound by 15-minute centrifugation at 4000 rpm and 37°C. All samples were analyzed by liquid chromatography with tandem mass spectrometry (LC-MS/MS) bioanalysis using ACQUITY UPLC, coupled with an Applied Biosystem Sciex 5500 triple quadrupole mass spectrometer. All data were acquired using Applied Biosystem Sciex 5500 and analyzed and processed using Analyst 1.7.2 software. Concentrations were back-calculated using an assay standard calibration curve in PBS and used for binding (maximal ligand binding capacity [B_max_] and ligand binding affinity [K_d_]) and stability calculations. Data were captured and processed using Analyst Version 1.7.2. Data were analyzed, and results were calculated using Microsoft Excel. Data sets were curve-fit by plotting the bound concentration versus the free concentration using GraphPad PRISM^®^ v.5.0 (one-site hyperbolic model).

### Computer modeling

Computer modeling of axitinib with TIE2 receptor was completed by CD ComputaBio (www.computabio.com, Shirley, NY). The protein structure for TIE2 was derived from the PDB database (PDBID: 2GY5) and force field parameters were taken from AMBER18 to build the molecular model for the receptor with tLEaP module in AMBER18. The structure for axitinib was built in GaussView and the model created by tLEaP module in AMBER18. The structure of the TIE2-axitinib complex was generated by docking axitinib in TIE2 with Autodock-VINA.

Computer modeling of vorolanib with VEGFR1, VEGFR2, and VEGFR3 was done by CD ComputaBio (www.computabio.com, Shirley, NY). The structures of VEGFR1 and VEGFR2 were derived from PDB database (PDBIDs 3HNG and 4AGD, respectively). As the structure of VEGFR3 has not been published, VEGFR1 and VEGFR2 were used as templates with homologous modeling using Modeller 10.4 software to obtain the predicted VEGFR3 structure. Vorolanib was docked with each of the three VEGFRs. Molecular dynamics was used to optimize the complexes, and binding energy was calculated after simulation by MMPBSA methods. From the three protein structures, AMBER14SB was used to calculate the charge of proteins and H++3 was used to distribute the pharmacokinetic values of amino acids. RDKit was used to generate a 3D structure of vorolanib, and MMFF94 was used to optimize structures and render the low-energy structure. UCSF Chimera was used to assign AM1-BCC local charge to vorolanib. The binding pocket was set as a quadrate box with 22.5 Å length, and the spacing step was 0.375. The maximum limit number of conformation search was set as 10,000. Genetic algorithm was applied for conformation sampling and scoring, and the optimal conformation was selected by docking scores. Molecular docking in simulation was performed using Autodock4.2.

To investigate the binding modes between vorolanib and VEGFR1, VEGFR2, and VEGFR3, Gromacs 5.1.5 was used to perform the molecular dynamics simulation. The simulation system was set as a closed environment with physiological temperature, pH 7.4, and a pressure of 1 bar. The periodic boundary setting of the simulation system was centered on the protein, and the minimum distance between the protein edge and the box edge was set as 0.1 nm. The GAFF force field was used for ligand atoms. TIP3P water molecules were added to simulate the water environment, and NaCl solvent was used to balance the system charge. After modeling the initial complex, the steepest descent method was applied to all atoms to minimize the energy in the system (i.e., constant number of particles, volume, and temperature [NVT] and constant number of particles, pressure, and temperature [NPT] equilibration under position restraints). After NVP and NPT equilibration, the systems were simulated for 50 ns. The covalent bond length was limited by linear constraint solving algorithm, and the long-distance electrostatic interaction was calculated using the particle mesh Ewald method. After all simulations were completed, Radius of Gyrate (Rg), hydrophobic contact calculation and root-mean-square deviation, root-mean-square fluctuation, etc., were performed using gmx module. Born and surface area solvation (MM/GBSA) methods were used to calculate binding energy (ΔG_bind_) between protein and molecule. The MMPBSA.py program integrated with AmberTools was used to calculate binding energy. Protein-ligand interaction analysis was completed using protein-ligand interaction profiler.

## Results

### Kinase screening assay

**Fig 2:**
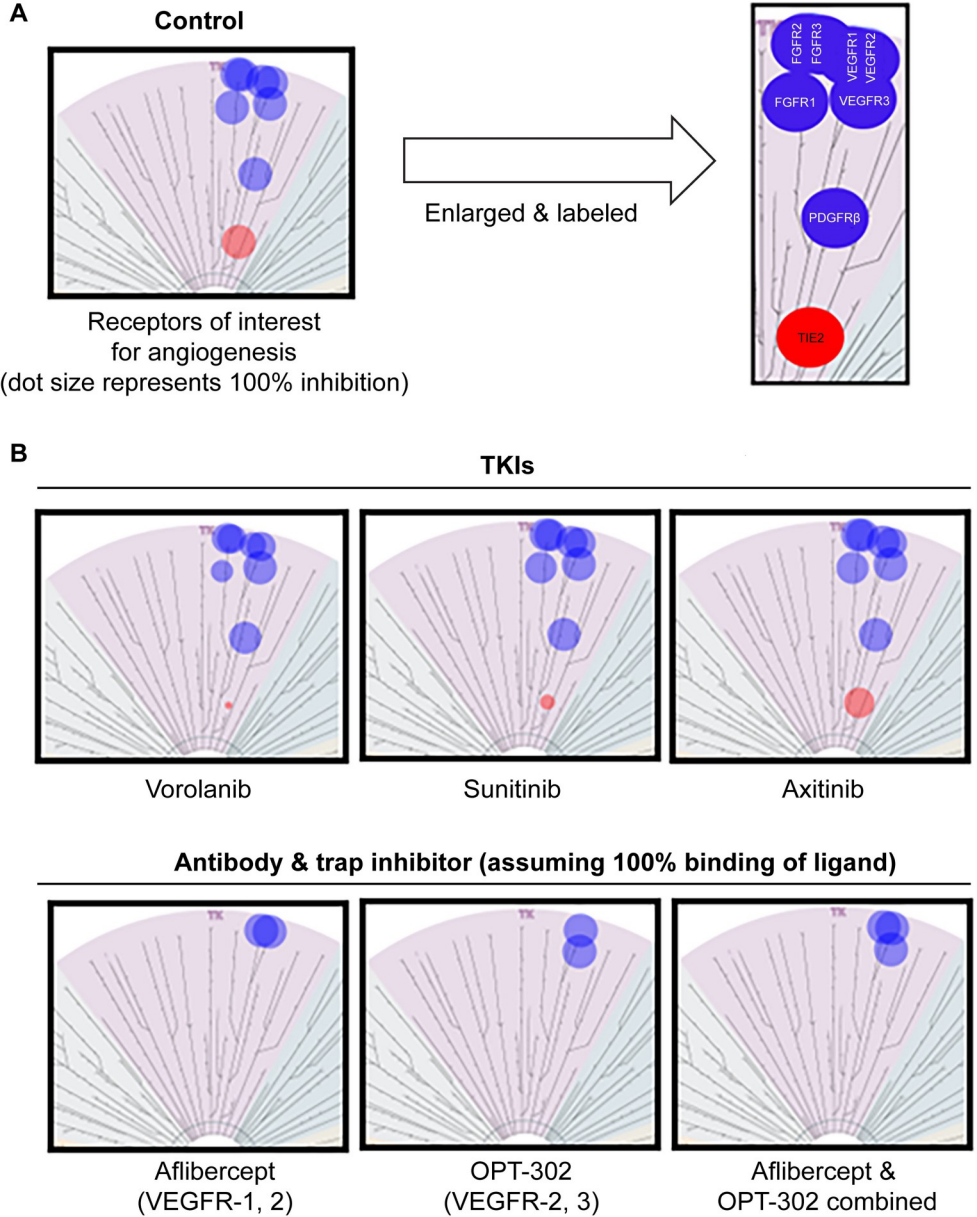
Kinase assay screen using 10μM of each TKI. (A) Control (baseline) TREEspot image depicting what 100% inhibition of all receptors associated with angiogenesis (blue) would look like. TIE2 receptor signaling is essential to maintain blood vessel stability and 100% inhibition of the TIE2 receptor is depicted (red). (B) In comparison to anti-VEGF antibodies, the three tested TKIs effectively inhibit all the receptors involved in pathological angiogenesis; the TKIs differ from each other as axitinib strongly inhibits TIE2, which is undesirable as maintained TIE2 function is essential for vascular stability. TKI, tyrosine kinase inhibitor; VEGF, vascular endothelial growth factor.

Multiple RTKs are involved in pathological angiogenesis including those in the VEGFR and FGFR families and PDGFRβ. To study these receptors, an in vitro kinase screen was performed that examined the ability of each TKI to inhibit each individual receptor. Primary screens for discovery research purposes are conducted at relatively high concentrations of compounds (e.g., (S1 Fig) concentrations of the three TKIs: vorolanib, sunitinib, and axitinib. The HotSpot™ in vitro kinase screen assay demonstrated that all three TKIs have potent (> 75%) pan-VEGFR inhibition (Fig 2). Axitinib appears to be a stronger inhibitor of FGFRs, but all three TKIs showed inhibition of PDGFRβ (> 90%), FGFR1 (> 65%), FGFR2 (> 90%), and FGFR3 (> 85%) at 10μM. Hence, in vitro, all three TKIs inhibited other RTKs known to be potentially associated with pathological angiogenesis.

One difference among the three TKIs pertained to inhibition of TIE2, a receptor responsible for maintaining vascular stability. Axitinib demonstrated potent inhibition of the TIE2 receptor at both and concentrations with 89% and 81% inhibition, respectively. In comparison, at and concentrations the TIE2 percentage inhibition by sunitinib (44% and 5%, respectively) and vorolanib (21% and 4%, respectively) was low and, in the case of vorolanib, negligible. For inhibition to be physiologically impactful it must be > 50% as supported by the fact that heterozygotes (lacking > 50% expression of a gene) have no phenotype. Based on the HotSpot™ kinase screening results, a secondary screen to determine the TIE2 IC_50_ for all three small molecules was completed.

### IC_50_ measurements

**Fig 3:**
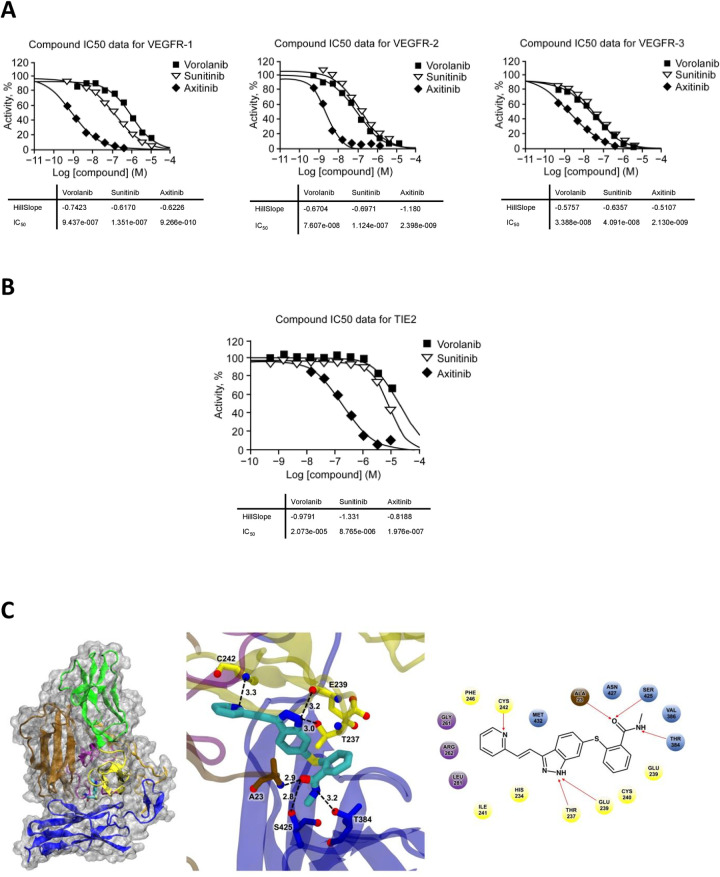
IC_50_ measurements. (A) IC_50_ curves for the three TKIs inhibiting VEGFR1, VEGFR2, and VEGFR3. (B) TIE2 IC_50_ curves for the three TKIs. The TIE2 IC_50_ confirmed the kinase screen data as the three tested TKIs differed greatly in their ability to inhibit TIE2 receptor. Note that all TKI compounds were prepared and tested in duplicate in a ten-dose IC_50_ model. (C) Computer modeling of axitinib with TIE2 receptor. On the left, the protein is displayed using ribbons while the protein surface is displayed using a white transparent pattern. The small molecule axitinib is shown as a teal stick image. The middle and left side of the figure shows the detailed analysis of the binding mode of small molecule simulation with TIE2 in a steady state. In the middle are 3D diagrams of interaction with the hydrogen bonds shown by a dotted line. On the right side is a 2D diagram depicting the axitinib interaction with hydrogen bonds indicated with a red arrow. IC_50_, half-maximal inhibitory concentration; TIE2, tyrosine kinase with immunoglobulin-like and EGF-like domains 2; TKI, tyrosine kinase inhibitor; VEGFR, vascular endothelial growth factor receptor.

VEGFR IC_50_ values were determined for each TKI (Fig 3A). The IC_50_ data supported the primary kinase screen findings and confirmed that vorolanib, sunitinib, and axitinib all have potent pan-VEGFR inhibition, with axitinib showing the strongest inhibition. To further validate the kinase screen findings, TIE2 IC_50_ values for all three TKIs were determined. The TIE2 IC_50_ results confirmed that axitinib is a potent TIE2 inhibitor, whereas vorolanib and sunitinib display IC_50_ values that are one to two orders of magnitude higher (Fig 3B). Computer modeling of TIE2 with axitinib predicts numerous hydrogen bonds being responsible for the strong binding complex (Fig 3C). Axitinib displayed strong positional stabilities and stable interactions with the receptor in the binding site. Key TIE2 residues for binding interactions between axitinib and TIE2 are predicted to be: T237, E239, C242, T383, S425, and N427.

Table 1 shows the TIE2 IC_50_ values determined in this study. Table 1 and S2 Fig provide observed and reported TKI levels in the retina and choroid of previously studied in vivo models treated with these TKIs at clinically relevant doses [34, 38–40]. Based on these combined data, we can conclude that vorolanib is a TKI that will not have a physiological impact on TIE2 receptor at any clinically relevant dose.

**Table 1:** Postulated TIE2 in vivo inhibition based on clinical TKI dosing and tested models. To understand the relevance of TIE2 inhibition, observed and reported TKI levels in retina/choroid of pre-clinical in vivo models treated with a clinical dosage of the TKIs were analyzed. The observed/reported TKI information in this table was extrapolated from the indicated references, which are publicly available presentations/publications, and that information did not provide statistical data. TIE2 IC_50_ values for each TKI were determined by Reaction Biology (US) and shown in Fig 3B.

TKI	Molecular weight (g/mol)	TIE2 IC_50_ (nM)	TIE2 IC_50_ (ng/g)	Species and observed/reported retina/choroid TKI level at ~600 μg dose	Approximate fold greater in species retina/choroid than TIE2 IC_50_
Vorolanib	439.5	20,730	9092	Rabbit < 300 ng/g [38]	Never greater
Sunitinib	398.5	8765	3492	NA	NA
Axitinib	386.5	197	76	Rabbit < 4000 ng/g [39] NHP < 4000 ng/g [34, 40]	> 50-fold in both rabbit and NHP

Observed and reported TKI levels in the retina and choroid of pre-clinical in vivo models treated with a clinical dosage of each TKI are also provided.

IC_50_, half-maximal inhibitory concentration; NA, not available; NHP, nonhuman primate; TIE2, tyrosine kinase with immunoglobulin-like and EGF-like domains 2; TKI, tyrosine kinase inhibitor.

### HUVEC sprouting assay

**Fig 4:**
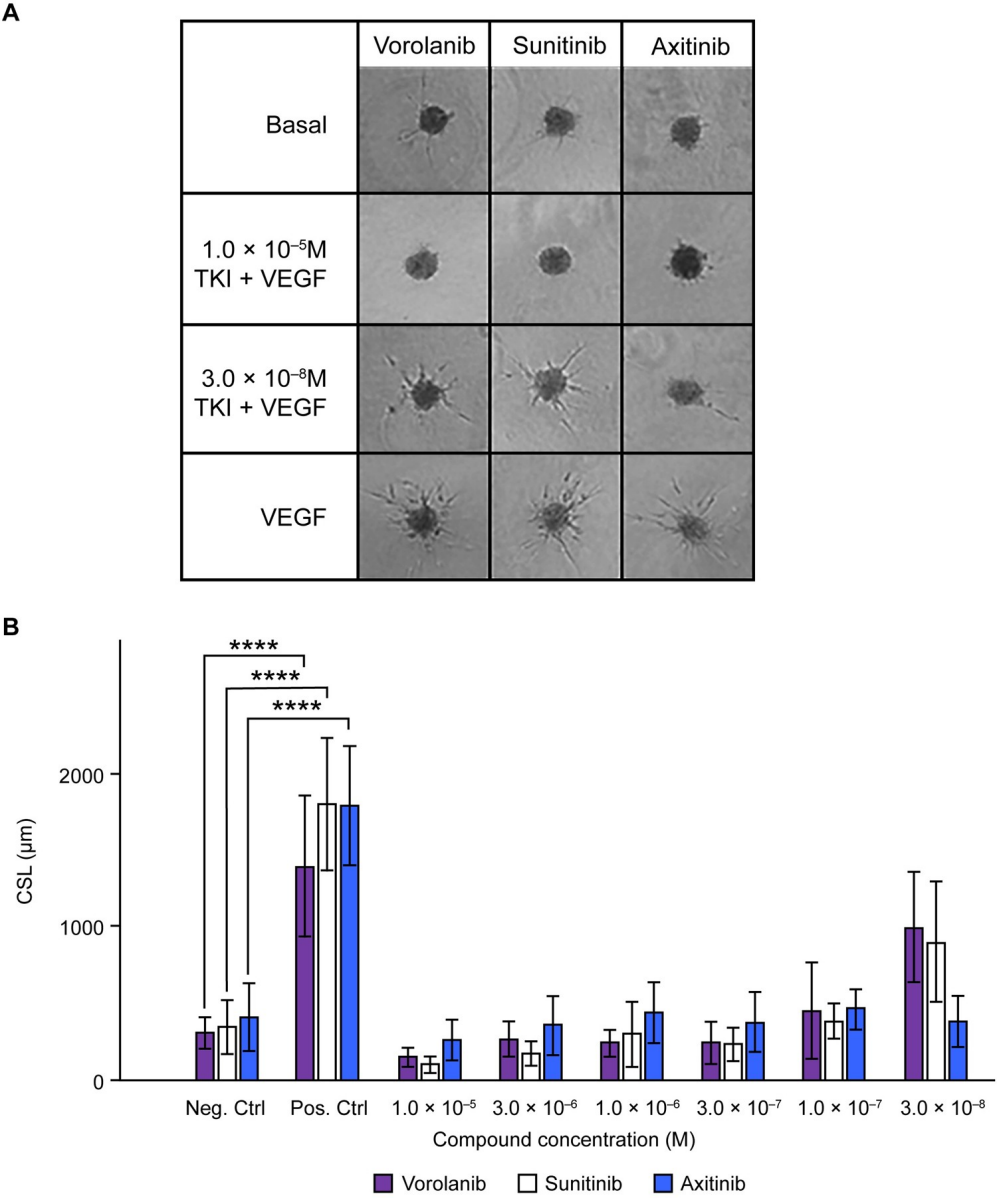
HUVEC sprouting assay results. (A) Representative photos of the spheroids when treated with two different concentrations of each TKI. (B) Quantitative analysis of the cumulative sprout length per spheroid (CSL) for each TKI at the different test concentrations. Results are (mean ± SD). Controls were ± 25 ng/mL VEGF-A. Results are (mean ± SD). Statistical differences were made visible by the presence of stars: *0.05 ≥ p > 0.01; **0.01 ≥ p > 0.001; ***0.001 ≥ p ≥ 0.0001; ****0.0001 ≥ p. CSL, cumulative sprout length; HUVEC, human umbilical vein endothelial cell; SD, standard deviation; TKI, tyrosine kinase inhibitor; VEGF, vascular endothelial growth factor.

At the highest test concentration of 1×10^–5^M, zero vessel sprouting was noted for vorolanib, sunitinib, and axitinib as evidenced by the representative spheroid images (Fig 4A). No statistical difference among the three TKIs was observed when they were tested in the range of 1.0×10^–5^M to 3.0×10^–7^M (Fig 4B), which is greater than the IC_50_ for all three. As expected, some sprouting was observed when the TKIs were applied at less than their IC_50_ value. The HUVEC sprouting assay confirmed that when utilized at a concentration near or greater than their IC_50_ value for VEGFR2, all three TKIs performed similarly and were effective in vitro inhibitors of VEGF-induced angiogenesis. These data also show that when any of the TKIs were used at concentrations that greatly exceed their IC_50_ value for VEGFR2, no further enhancement in inhibition of HUVEC vessel sprouting was achieved.

### CAM assay

**Fig 5:**
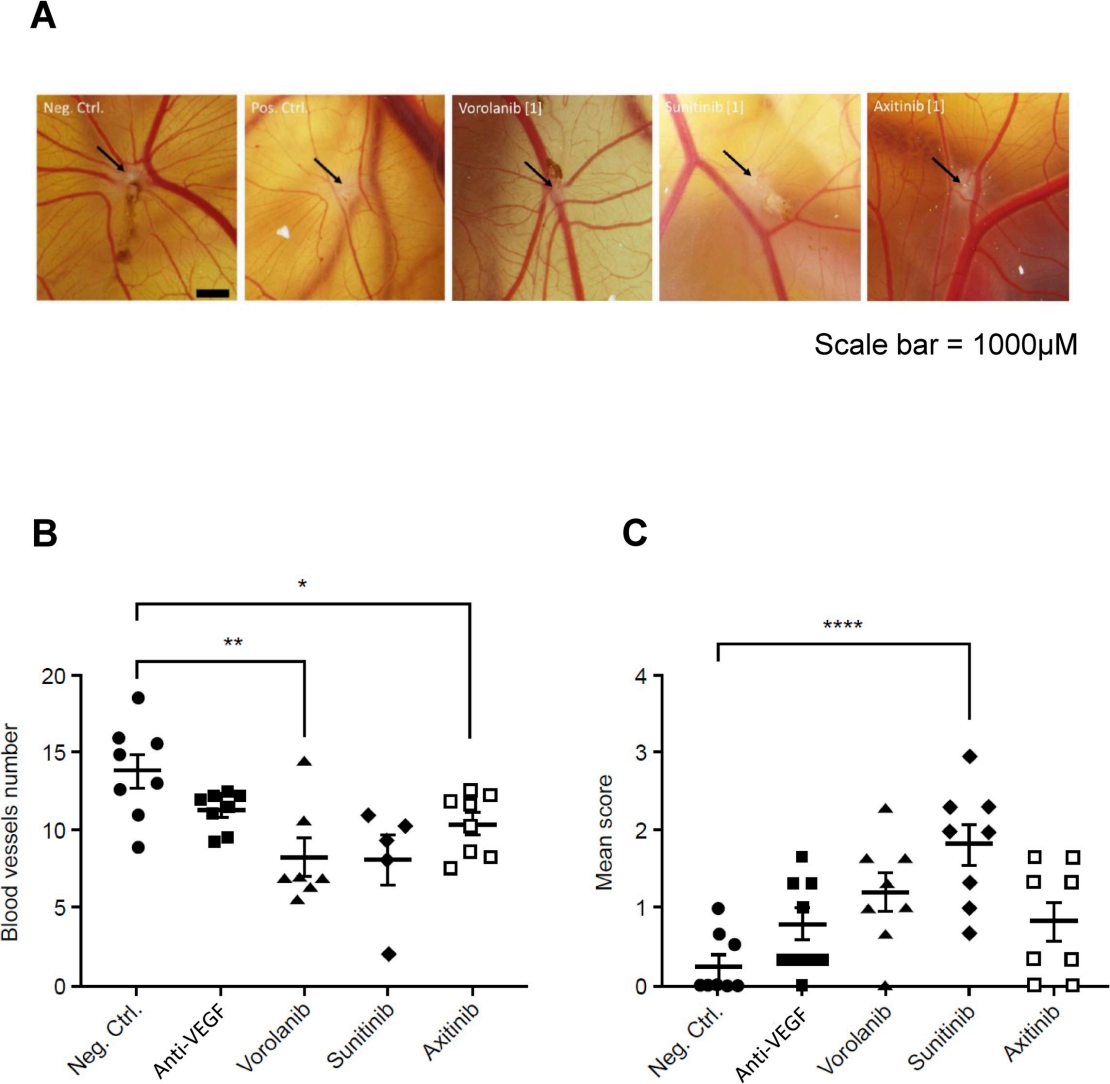
CAM assay results for the three TKIs tested at their VEGFR2 IC_50_ value (vorolanib = 52nM, sunitinib = 43nM, and axitinib = 0.2nM). (A) Representative images of gelatin sponge and surrounding vessels in ovo for negative control, positive control (anti-VEGF antibody, bevacizumab) and the three tested TKIs. Black arrows point at the white spot which is the location of the gelatin sponge. Scale bar is 1000 μm. (B) The number of macroscopic blood vessels perfusing the gelatin sponge for each TKI was determined (mean ± SEM). (C) The individual semi-quantitative scoring of vessel size for each TKI is shown (mean ± SEM). For panels B and C, statistical differences were made visible by the presence of stars: *0.01 < p ≤ 0.05; **0.001 < p ≤ 0.01; ***0.0001 ≤ p ≤ 0.001; ****p ≤ 0.0001. CAM, chorioallantoic membrane; IC_50_, half-maximal inhibitory concentration; SEM, standard error of the mean; TKI, tyrosine kinase inhibitor; VEGFR, vascular endothelial growth factor receptor.

The anti-angiogenic activity of each TKI was evaluated by counting the number of vessels perfusing a gelatin sponge implanted on top of a CAM in ovo. Fig 5A shows representative photographs of sponges and surrounding vessels when the TKIs were utilized at their individual VEGFR2 IC_50_ values as well as photographs for the negative control (PBS/DMSO vehicle) and positive control (anti-VEGF antibody, bevacizumab). The reference compound (positive control) was bevacizumab (AVASTIN^®^), a known anti-VEGF antibody that possesses anti-angiogenic properties and has been shown to decrease the number of nodes and branching in the CAM assay [41]. Bevacizumab led to a 18% vessel reduction compared to the negative control with p = 0.05. Bevacizumab tends to influence vessel diameter as evidenced by an increase in vessel diameter score compared to the negative control (p = 0.07) (Fig 5B).

All three TKIs were found to inhibit angiogenesis to a greater degree than bevacizumab as evidenced by mean values (Fig 5B). Relative to the negative control, only vorolanib showed statistically significant inhibition at both its IC_50_ and 200× IC_50_ doses, reducing the number of blood vessels perfusing the sponges by 39.9% and 37.1%, respectively (Figs 5B and S3). Sunitinib significantly inhibited angiogenesis relative to the negative control only at its 200× IC_50_ dose by 49.3% versus vehicle (Figs 5B and S3). Though IC_50_ data indicated that axitinib more potently inhibited the VEGFRs, the in vivo work showed that axitinib only exhibited statistically significant inhibition of angiogenesis relative to the negative control at its IC_50_ dose by 24.6% versus vehicle (Figs 5B and S3).

Using semi-quantitative scoring, a higher mean percentage of blood vessels reaching the sponge had standard diameter when treated with doses of vorolanib or sunitinib at IC_50_ versus the bevacizumab positive control (Fig 5C). The impact of IC_50_ dose of axitinib on vessel diameter was less apparent as the mean score for axitinib was similar to bevacizumab (Fig 5C).

### Melanin-binding assay

Both vorolanib and axitinib showed little or no binding to melanin, so the K_d_ and B_max_ values of these two TKIs could not be determined (Table 2). In comparison, sunitinib bound melanin with a K_d_ of 13.5μM and a B_max_ of 302 nmol/mg melanin. The results of the melanin-binding assay identify sunitinib as the only TKI of the three tested to bind melanin.

**Table 2:** Melanin-binding data using a 0.06 to 25.0μM concentration range for all TKIs. Sunitinib was the only TKI of the three tested TKIs to exhibit definitive characteristics of melanin binding. SEM is indicated in the table.

Compound	K_d_ (μM)	B_max_ (nmol/mg melanin)	Mean % bound (across all concentrations)
**Vorolanib**	ND	ND	14.3
**Sunitinib**	13.5 ± 10.9	302	46.7
**Axitinib**	ND	ND	4.1
**Chloroquine**	3.06 ± 0.6	153	56.7

B_max,_ maximal ligand binding capacity; K_d,_ binding affinity; ND, not determined.

### Computer modeling of vorolanib with VEGFRs

**Fig 6:**
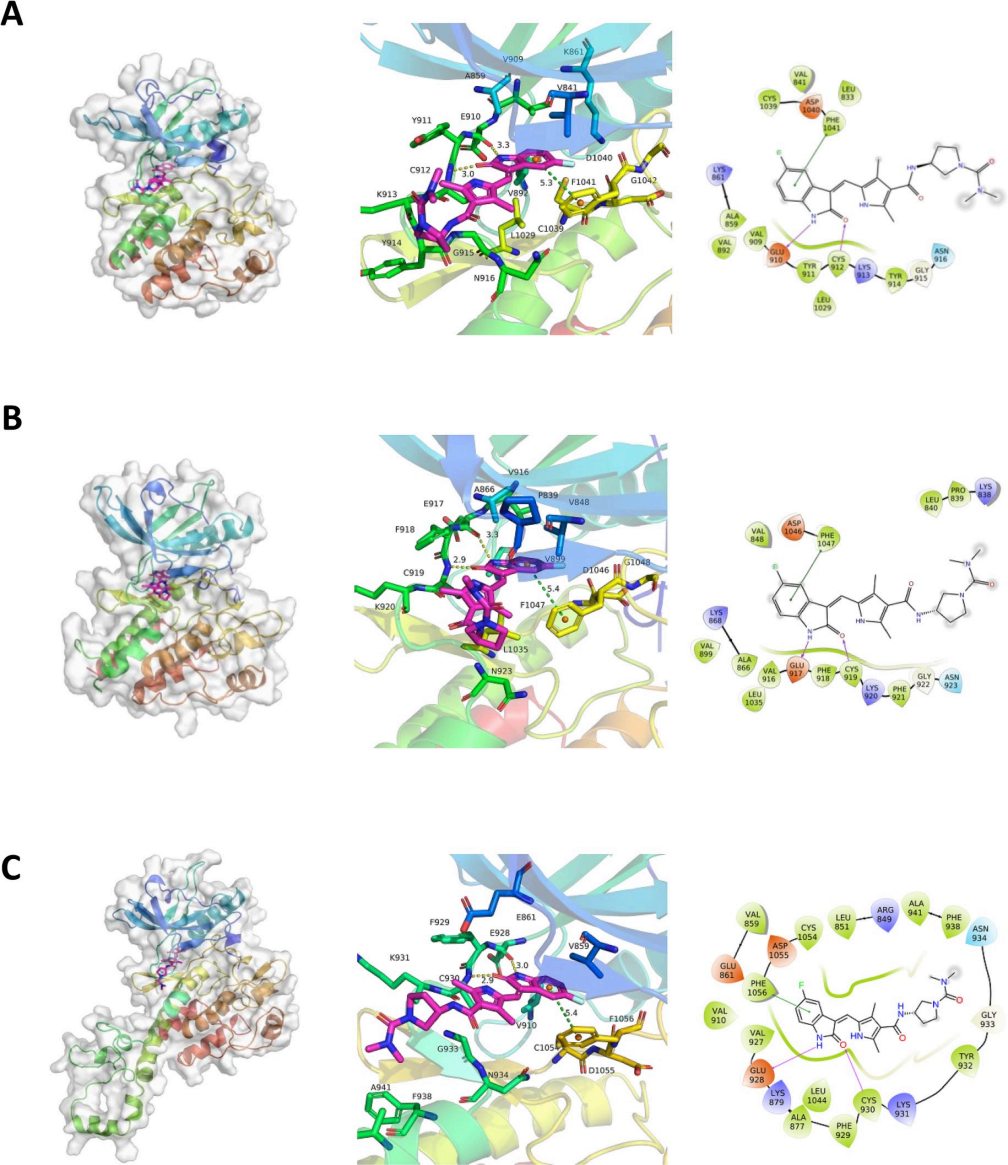
Computer modeling of vorolanib bound to VEGFRs. (A) VEGFR1; (B) VEGFR2; and (C) VEGFR3. Left panels, protein displayed using ribbons while the protein surface is displayed using a white transparent pattern. Vorolanib is shown as a purple stick image and image is at 50 ns stimulation. Left and center panels, binding of vorolanib with VEGFR at 50 ns stimulation. Center panels, 3D diagrams of interaction with hydrogen bonds indicated by yellow dotted lines and π-π stacking is indicated by green dotted lines. Right panels, 2D diagrams depicting the interaction of vorolanib with VEGFRs. Hydrogen bonds are indicated using purple arrows, and π-π stacking is indicated with a green line. VEGFR, vascular endothelial growth factor receptor.

The interactions of sunitinib and axitinib with VEGFRs have been modeled previously [34], but this had not been done with vorolanib. In this study, during dynamic simulation, vorolanib was always bound to the VEGFR1 kinase domain without breaking away from the protein, suggesting strong and stable binding. There were at least two hydrogen bonds between vorolanib and VEGFR1 and one π-π stacking during the simulation (Fig 6A). The phenyl group in vorolanib formed π-π stacking with VEGFR1 phenylalanine Phe1041 in the Asp-Phe-Gly "out" (DFG-out) conformation, which is the inactive form of VEGFR1. The amino group in the lactam bond of vorolanib formed a hydrogen bond with Glu910 and Cys912 in the hinge region of VEGFR1 with a retention rate of 99% and 98%, respectively, indicating that the binding to these two amino acids in the hinge region is very stable. The fluorobenz group of vorolanib formed π-π stacking with Phe1041, and this configuration was favorable for binding when VEGFR1 was in a DFG-out conformation. Glu910 and Cys912 can bind ATP; therefore, vorolanib occupied part of the ATP-binding pocket. This molecular modeling therefore suggests that vorolanib is a type II inhibitor because it bound VEGFR1 in the DFG-out conformation.

Computer modeling predicts that the binding of vorolanib to VEGFR2 is similar to vorolanib binding to VEGFR1 as vorolanib binds the ATP-binding pocket of VEGFR2 in the inactive DFG-out configuration. Vorolanib formed a very stable hydrogen bond with Glu917 and Cys919, and the distance between the hydrogen bond acceptor and donor was 3.3 Å and 2.9 Å, respectively (Fig 6B). For VEGFR2, additional stability occurs with the benzene ring of vorolanib forming π-π stacking with Phe1047 at a distance of 5.4 Å.

The solved structure of VEGFR3 was not available, so it was constructed for this study. Its predicted structure was found to be similar to VEGFR1 and VEGFR2 (Fig 6C). The amino group in the cyclo-lactam bond of vorolanib formed hydrogen bonds with Glu928 and Cys930 in the hinge region of VEGFR3, and the retention rates were 100% and 98%, respectively. Molecular modeling suggested that vorolanib bound to the kinase domain of VEGFR3, and this binding did not break during simulation, indicating strong binding. The modeling results for VEGFR3 suggested that the binding of vorolanib to these two amino acids in the hinge region has strong affinity and is very stable. As with the other two VEGFR family members, vorolanib bound VEGFR3 in the DFG-out configuration.

In these molecular dynamics simulations, the stereochemistry of vorolanib changed from S to R as the R configuration appeared to be the preferred shape for binding. This change is common in computational simulations.

## Discussion

**Fig 7:**
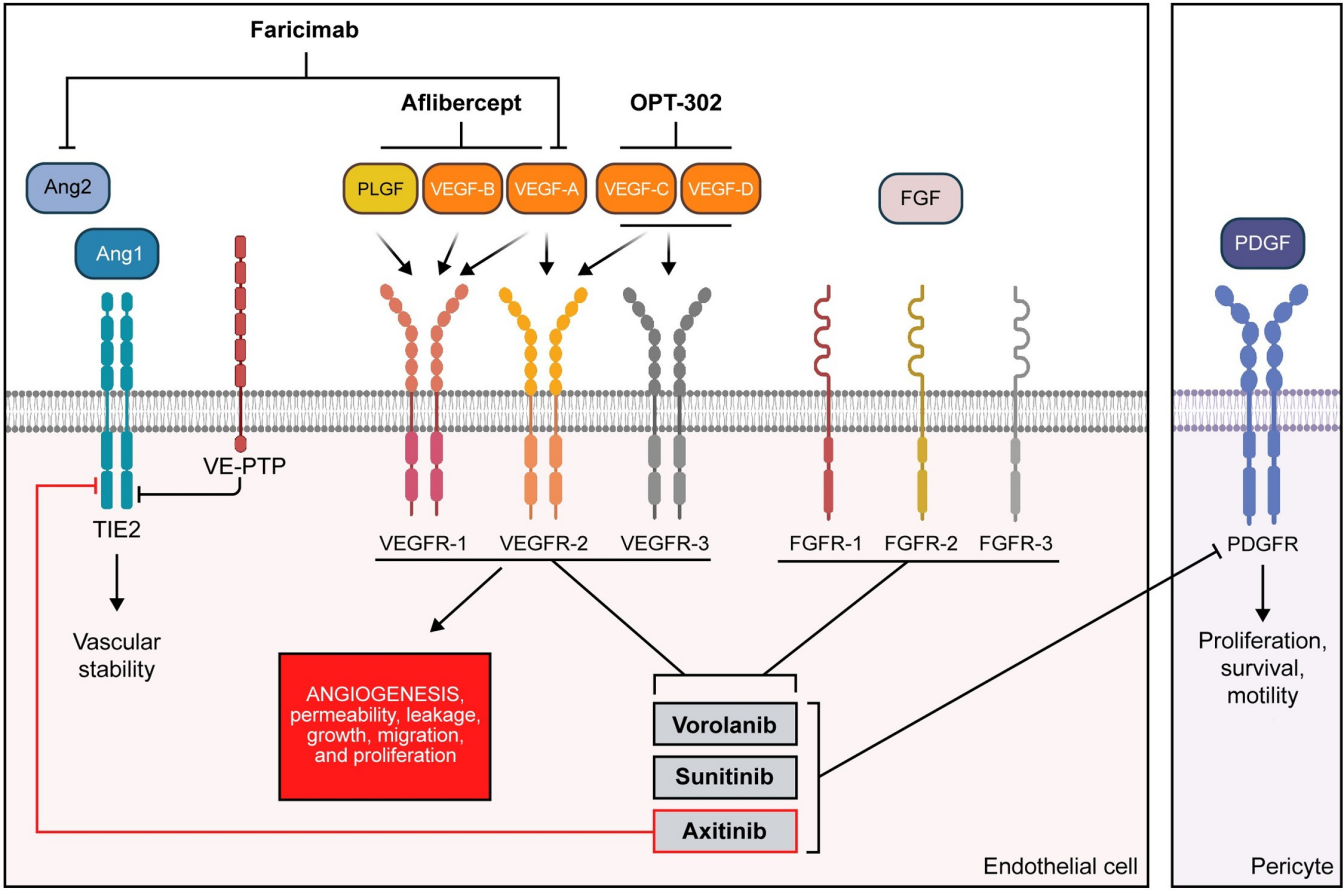
Schematic of cell membrane RTKs involved in angiogenesis and vascular stability, including stimulating ligands, anti-VEGF antibody therapies, and TKIs. All three TKIs showed pan-VEGFR inhibition and effectively inhibited all the receptors that participate in pathological angiogenesis. Axitinib was the only TKI identified as a potent inhibitor of TIE2 which is not desired as normal TIE2 function is essential as it functions to maintain vascular stability. This image was created using BioRender software. Ang, angiopoietin; FGFR, fibroblast growth factor receptor; PDGFR, platelet-derived growth factor receptor; PLGF, placental growth factor; RTK, receptor tyrosine kinase; TIE2, tyrosine kinase with immunoglobulin-like and EGF-like domains 2; TKI, tyrosine kinase inhibitor; VE-PTP, vascular endothelial cell-specific protein tyrosine phosphatase; VEGFR, vascular endothelial growth factor receptor.

Angiogenesis is a complicated process that involves multiple signaling pathways [42]. Pathological angiogenesis occurs in diseases such as cancer and wAMD. Our kinase screen results found that vorolanib, sunitinib, and axitinib strongly inhibit RTKs of the VEGFR and FGFR families as well as PDGFRβ. All these receptors are of great interest because they contribute to pathological angiogenesis. The current anti-VEGF antibody treatments used to inhibit angiogenesis in patients with wAMD only bind one or two VEGF ligands (Fig 7), which limits their impact on the disease because other ligands (e.g., VEGF-C, VEGF-D), receptors, and pathways participating in angiogenesis remain active. Pan-VEGF inhibition as offered by TKI treatment provides a more encompassing suppression of the signaling pathways involved in angiogenesis.

Of the VEGFR family members, VEGFR2 is viewed as the most critical in angiogenesis; therefore, its inhibition is essential for the treatment of VEGF-driven diseases [43]. TKIs differ in their potency for each individual RTK. Sunitinib IC_50_ values were previously reported to be 10nM for VEGFR1, VEGFR2, and VEGFR3 [44], but also as 17.25nM [21] and 43nM [45] for VEGFR2. For axitinib, IC_50_ values have been reported to be 0.1nM for VEGFR1, 0.2nM for VEGFR2, and 0.1 to 0.3nM for VEGFR3 [46]; however, another study reported a VEGFR2 IC_50_ of 7.3nM for axitinib [47]. Vorolanib IC_50_ values for VEGFR2 have been reported as 1.12nM [21] and 52nM [45]. The variability in IC_50_ values is attributed primarily to differences in assay conditions (e.g., choice of substrate, enzyme, or ATP concentration in reaction mixture) or use of generalized methods instead of optimized methods for individual receptors. The IC_50_ protocols that we utilized in our current study were general methods and not fully optimized to any individual RTK. As a result, our IC_50_ values for vorolanib, sunitinib, and axitinib do vary from the anticipated values that have been reported in some citations. For example, the VEGFR2 IC_50_ value of 2.3nM for axitinib that we observed was more than 10× greater than the reported value of 0.2nM [46] and more than 100× greater than the reported IC_50_ value of 0.02nM [48]. Regardless of the variability in our observed IC_50_ values for the VEGFRs, the IC_50_ values we obtained for all three TKIs are comparable to previous citations and remain at or below the levels expected in the retina or choroid at the therapeutic doses used in the clinical setting (e.g., EyePoint Pharmaceuticals, EYP-1901; Ocular Therapeutix, OTX-TKI). Although TKIs differ in their potency for each individual receptor, the IC_50_ data we obtained for vorolanib, sunitinib, and axitinib all support our primary kinase screening data and confirm that all three of these TKIs are strong pan-VEGFR inhibitors and effectively inhibit receptors that signal in pathological angiogenesis.

Our kinase screen identified axitinib as the only TKI in this study that is a potent TIE2 inhibitor, and this finding was confirmed by IC_50_ measurements. TIE2 inhibition is undesirable because this receptor is essential for maintaining vascular stability. Normal TIE2 functioning is essential for vascular stability and pericyte TIE2 controls sprouting angiogenesis [49]. Without TIE2 signaling due to its inhibition, pericyte loss and endothelial dysfunction could occur which would result in vascular destabilization which eventually progresses to vessel leakage. A kinase screen with axitinib has previously been reported in the literature [48] and in that screen, the investigators failed to identify axitinib as a TIE2 binder because their screen was conducted using axitinib at an extremely low 1nM concentration, considerably below the recommended screening concentration of 10μM [50].

The HUVEC sprouting assay is an in vitro method that was conducted to determine the ability of the three TKIs to effectively inhibit VEGF-induced angiogenesis. All three effectively inhibited vessel sprouting when applied at a concentration greater than or equal to their respective VEGFR2 IC_50_ values. This experiment demonstrates that in a VEGF-dependent study, any of the three TKIs when used at the VEGFR2 IC_50_ value will achieve the desired efficacy and when levels higher than IC_50_ are utilized the result is little to no enhancement in the effect; hence, the higher potency for VEGFR2 inhibition reported for axitinib relative to vorolanib and sunitinib appears to be of no benefit when compounds are present at concentrations at or above the receptor IC_50_ value for that particular TKI.

The angiogenic activity of each TKI was also evaluated in vivo using the CAM assay. All three TKIs inhibited angiogenesis more effectively than bevacizumab, suggesting that pan-VEGFR inhibition and suppression of multiple signaling pathways involved in angiogenesis is more effective than simply targeting and inhibiting a limited number of VEGF ligands. Of the three TKIs tested, only vorolanib exhibited statistically significant differences from the negative control at both IC_50_ and 200× doses. The results of the CAM assay substantiate the findings from the HUVEC assay as they demonstrate that once the IC_50_ value is met, then desired efficacy occurs, and the higher potency reported for axitinib relative to vorolanib and sunitinib does not provide additional benefit. Vorolanib and sunitinib prevented variation from the standard vessel diameter, whereas axitinib did not, which could potentially be related to the inhibition of TIE2 by axitinib; however, future investigation is necessary to determine the precise reason for this finding pertaining to axitinib. Normal TIE2 functioning is essential for vascular stability, and TIE2 in pericytes controls vessel sprouting [49]. If TIE2 signaling is inhibited, pericyte loss and endothelial dysfunction can occur, resulting in vascular destabilization that may eventually progress to vessel leakage and other associated AEs [29–31].

In the eye, melanin is present in the iris, choroid, and RPEs, and turnover in some of these ocular tissues is thought to be absent [23, 30]. Melanin binding by a drug is not predictive of retinal toxicity [24, 31], but tissue accumulation can be a concern if accumulated drug impacts normal cell function. Of the three TKIs we studied, only sunitinib bound melanin and the significance of this binding is an area requiring future investigation. The absence of melanin binding by vorolanib and by axitinib reduces potential safety concerns for these two compounds because their tissue accumulation due to melanin-binding is unlikely.

To gain greater insight into the mode of action of vorolanib and identify the TKI class to which it belongs, we modeled the binding of vorolanib with each VEGFR. Sunitinib is a type I½B/II½B kinase inhibitor while axitinib is a type IIA inhibitor [51]. Type I½ inhibitors such as sunitinib bind to the active DFG-in kinase conformation, whereas type II inhibitors such as axitinib bind to the inactive DFG-out conformation [51]. Type I, I½, and II inhibitors occupy part of the ATP binding pocket and form hydrogen bonds [51] with the hinge region of the RTK. Sunitinib is considered an outlier because it does not bind in the region of the ATP pocket but rather has interactions outside the ATP binding site [48]. Type I, I½, and II inhibitors are further divided into A and B subtypes with the subtype A inhibitors binding the region separating the small and large lobes of the protein while binding of subtype B inhibitors does not extend into the back cleft of this region [52]. Roskoski suggested that subtype A inhibitors act for an extended time (minutes to hours) while subtype B inhibitors act for a short duration (seconds to minutes) [52]. Our modeling results predict that vorolanib strongly binds intracellularly near the ATP binding regions of VEGFR1, VEGFR2, and VEGFR3 when the VEGFRs are in the DFG-out (inactive) configuration, making vorolanib a type II pan-VEGFR inhibitor. It is advantageous for a TKI to be type II because it means enhanced specificity versus type I inhibitors [53]. The results of the computer modeling work enhance our understanding of the mechanism of action of vorolanib and explain its potency as a pan-VEGFR inhibitor. The finding that vorolanib is likely a type II TKI differentiates it from sunitinib, from which vorolanib was derived [20].

Angiogenesis involves multiple signaling pathways (Fig 7). The data from our studies suggest that pan-VEGFR TKIs may have advantages over the anti-VEGF antibodies currently prescribed for ocular diseases such as wAMD. In the in vitro HUVEC assay, all three TKIs effectively inhibited angiogenesis. In the in vivo CAM assay, the mean values observed for all three TKIs were better than the positive control anti-VEGF antibody, meaning that the TKIs inhibited angiogenesis effectively. Computer modeling of vorolanib provides insight into its mode of action and predicts that vorolanib is a type II inhibitor. Type II inhibitors offer more specificity than type I inhibitors [53]. In this study, vorolanib was found to differentiate itself from both sunitinib and axitinib as vorolanib does not bind melanin nor does it inhibit TIE2. With this study, we have enhanced our understanding of the mechanism of action of vorolanib and distinguished the TKIs from anti-VEGF antibodies as well as from each other. Vorolanib was found to be an effective pan-VEGFR inhibitor that effectively inhibits receptors known to signal in pathological angiogenesis such as observed in wAMD, without evidence of compromising vascular stability by inhibiting TIE2. Intravitreal delivery of a pan-VEGFR TKI such as vorolanib (Fig 7) may provide benefits that currently approved therapies cannot offer and address unmet medical needs for patients with wAMD.

## Supporting information

S1 Fig Results of kinase assay screen. Kinase assay screen using 1μM of each TKI was performed by Reaction Biology Corporation (US). Control (baseline) TREEspot images depicts what 100% inhibition of all receptors associated with angiogenesis (blue) would look like. TIE2 is necessary to maintain blood vessel stability and the control image depicts what 100% inhibition of the TIE2 receptor would look like (red). TIE2, tyrosine kinase with immunoglobulin-like and EGF-like domains 2; TKI, tyrosine kinase inhibitor.
(PDF)

S2 Fig Plot of TKI concentrations post-injection that have been observed/reported in the retina/choroid of pre-clinical in vivo models. VEGFR2 IC_50_ value where VEGFR2 IC_50_ for vorolanib (52nM; 23 ng/mL) and axitinib (0.2nM; 0.07 ng/mL). Data sources are provided in Table 1. IC50, half-maximal inhibitory concentration; TKI, tyrosine kinase inhibitor; VEGFR, vascular endothelial growth factor receptor.
(PDF)

S3 Fig CAM assay results for the three TKIs tested at a 200× VEGFR2 IC_50_ value. VEGFR2 IC_50_ for each TKI was: vorolanib (52nM), sunitinib (43nM) and axitinib (0.2nM). The number of macroscopic blood vessels perfusing the gelatin sponge for each TKI was determined (mean ± SEM). Statistical differences were made visible by the presence of stars: *0.05 ≥ p > 0.01; **0.01 ≥ p > 0.001; ***0.001 ≥ p ≥ 0.0001; ****0.0001 ≥ p. CAM, chorioallantoic membrane; SEM, standard error of the mean; TKI, tyrosine kinase inhibitor; VEGFR, vascular endothelial growth factor receptor.
(PDF)

S1 Dataset (XLSX)

S1 File (PDF)
