# Supplementary material for: Vorolanib, sunitinib, and axitinib: A comparative study of vascular endothelial growth factor receptor inhibitors and their anti-angiogenic effects
Source: PLoS One. 2024 Jun 4;19(6):e0304782. doi: 10.1371/journal.pone.0304782 (PMC11149885; doi:10.1371/journal.pone.0304782)

S1 |Fig. Results of kinase assay screen. Kinase assay screen using 1 μM of each TKI was performed by Reaction Biology Corporation. Control (baseline) TREEspot images depicts what 100% inhibition of all receptors associated with angiogenesis (blue) would look like. TIE2 is necessary to maintain blood vessel stability and the control image depicts what 100% inhibition of the TIE2 receptor would look like (red).

TIE2, tyrosine kinase with immunoglobulin-like and EGF-like domains 2; TKI, tyrosine kinase inhibitor.

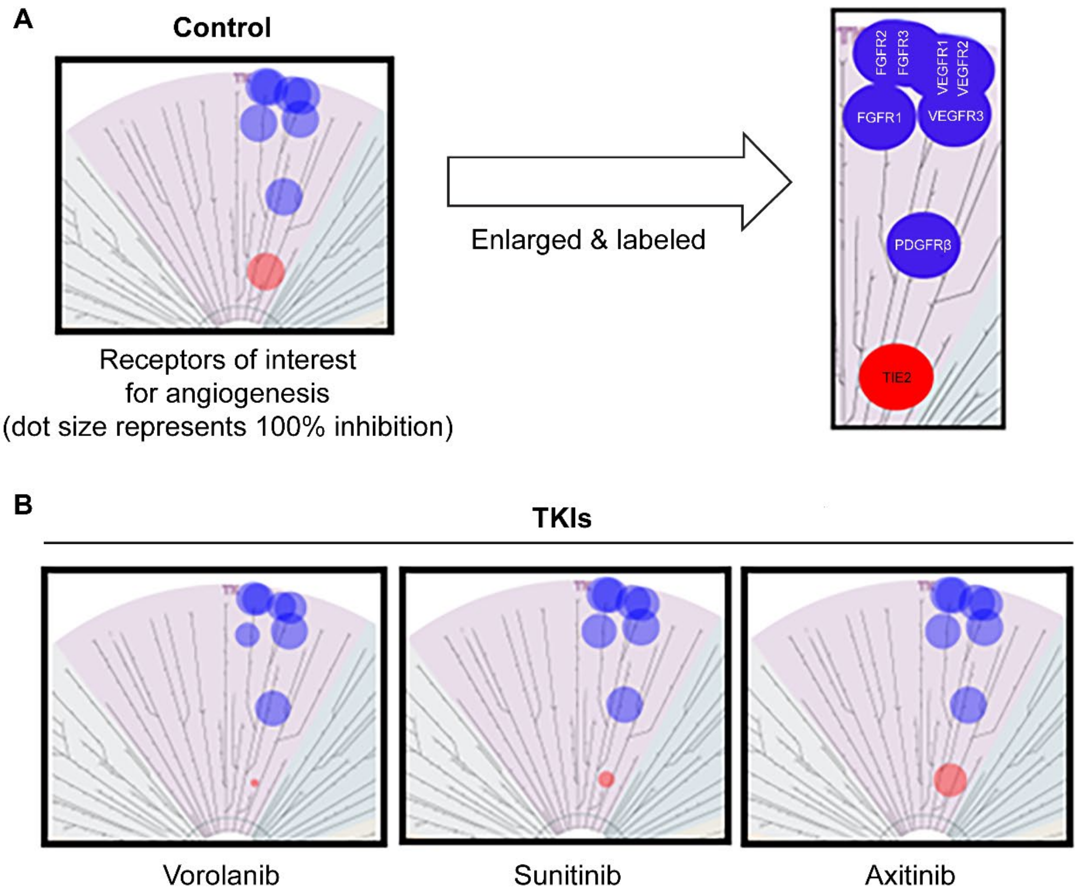

Supplement: S1 Fig — Kinase assay screen using 1μM of each TKI was performed by Reaction Biology Corporation (US). Control (baseline) TREEspot images depicts what 100% inhibition of all receptors associated with angiogenesis (blue) would look like. TIE2 is necessary to maintain blood vessel stability and the control image depicts what 100% inhibition of the TIE2 receptor would look like (red). TIE2, tyrosine kinase with immunoglobulin-like and EGF-like domains 2; TKI, tyrosine kinase inhibitor. (PDF) [file pone.0304782.s001.pdf]
