# Supplementary material for: Vorolanib, sunitinib, and axitinib: A comparative study of vascular endothelial growth factor receptor inhibitors and their anti-angiogenic effects
Source: PLoS One. 2024 Jun 4;19(6):e0304782. doi: 10.1371/journal.pone.0304782 (PMC11149885; doi:10.1371/journal.pone.0304782)

S2 Fig. Plot of TKI concentrations post-injection that have been observed/reported in the retina/choroid of pre-clinical in vivo models. Data sources are provided in Table 1.

TKI, tyrosine kinase inhibitor.

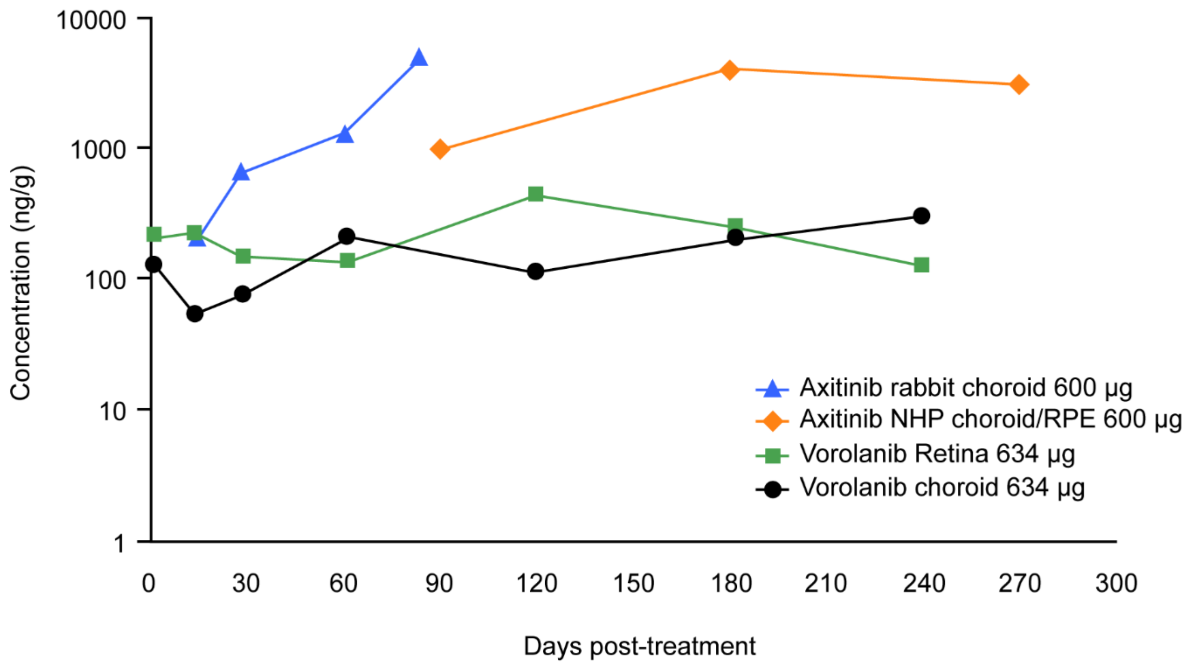

Supplement: S2 Fig — VEGFR2 IC50 value where VEGFR2 IC50 for vorolanib (52nM; 23 ng/mL) and axitinib (0.2nM; 0.07 ng/mL). Data sources are provided in Table 1. IC50, half-maximal inhibitory concentration; TKI, tyrosine kinase inhibitor; VEGFR, vascular endothelial growth factor receptor. (PDF) [file pone.0304782.s002.pdf]
