# Supplementary material for: Vorolanib, sunitinib, and axitinib: A comparative study of vascular endothelial growth factor receptor inhibitors and their anti-angiogenic effects
Source: PLoS One. 2024 Jun 4;19(6):e0304782. doi: 10.1371/journal.pone.0304782 (PMC11149885; doi:10.1371/journal.pone.0304782)

S3 Fig. CAM assay results for the three TKIs tested at a 200X VEGFR-2 IC_{50} value. The number of macroscopic blood vessels perfusing the gelatin sponge for each TKI was determined (mean ± SEM). The concentration of compound in the injection is shown. The injection volume was 10 µL in each case. Statistical differences were made visible by the presence of stars: *0.05 ≥ p > 0.01; **0.01 ≥ p > 0.001; ***0.001 ≥ p ≥ 0.0001; ****0.0001 ≥ p.

CAM, chick chorioallantoic membrane; SEM, standard error of the mean; TKI, tyrosine kinase inhibitor; VEGFR, vascular endothelial growth factor receptor.

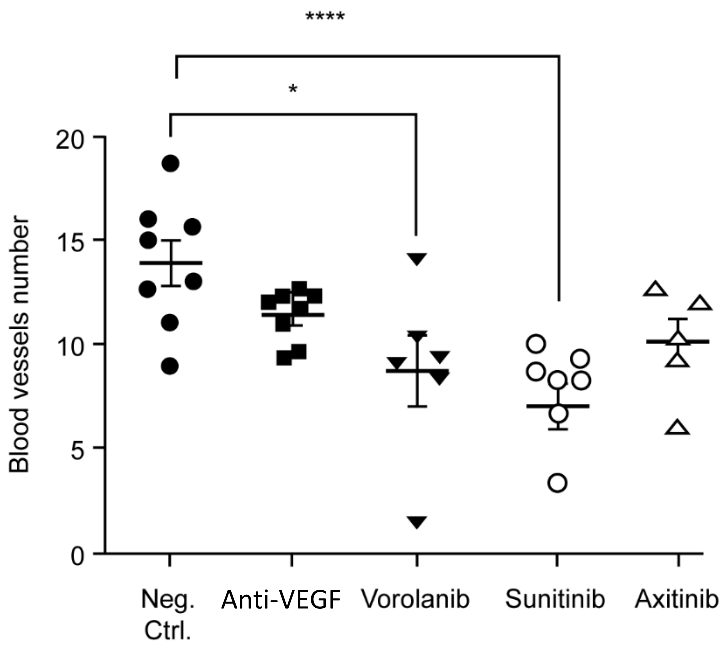

Supplement: S3 Fig — VEGFR2 IC50 for each TKI was: vorolanib (52nM), sunitinib (43nM) and axitinib (0.2nM). The number of macroscopic blood vessels perfusing the gelatin sponge for each TKI was determined (mean ± SEM). Statistical differences were made visible by the presence of stars: *0.05 ≥ p > 0.01; **0.01 ≥ p > 0.001; ***0.001 ≥ p ≥ 0.0001; ****0.0001 ≥ p. CAM, chorioallantoic membrane; SEM, standard error of the mean; TKI, tyrosine kinase inhibitor; VEGFR, vascular endothelial growth factor receptor. (PDF) [file pone.0304782.s003.pdf]
