# Supplementary material for: Vorolanib, sunitinib, and axitinib: A comparative study of vascular endothelial growth factor receptor inhibitors and their anti-angiogenic effects
Source: PLoS One. 2024 Jun 4;19(6):e0304782. doi: 10.1371/journal.pone.0304782 (PMC11149885; doi:10.1371/journal.pone.0304782)
Supplement: S1 File — (PDF) [file pone.0304782.s005.pdf]

ARVO 2024

View Abstract

CONTROL ID: 4049203

SUBMISSION ROLE: Abstract Submission

AUTHORS

AUTHORS (LAST NAME, FIRST NAME): Lynch, Jeff[1]; Bakri, Sophie J.[2]; Howard-Sparks, Michelle[1]; Saint-Juste, Stephan[3]; Saim, Said[1]

INSTITUTIONS (ALL): 1. EyePoint Pharmaceuticals, Inc., Watertown, PA, United States.
2. Ophthalmology, Mayo Clinic Minnesota, Rochester, MN, United States.
3. Department of Polymer Science and Engineering, University of Massachusetts Amherst, Amherst, MA, United States.

Commercial Relationships Disclosure: Jeff Lynch: Commercial Relationship(s);Code E (Employment):EyePoint Pharmaceuticals, Inc. | Sophie Bakri: Commercial Relationship(s);Code C (Consultant/Contractor):Abbvie, Adverum, Allergan, Amgen, Annexon, Apellis, Aviceda, Cholgene, Eyepoint, ilumen, Iveric bio, Kala, Genentech, Neurotech, Novartis, Ocular Therapeutix, Outlook, Pixium, Regenxbio, Regeneron, Rejuvitas, Revana, Roche, VoxelCloud, Zeiss;Code S (non-remunerative):Serves on the board of the American University Professors of Ophthalmology.;Code F (Financial Support):Her institution received research grants from Lowy Medical Foundation and Regenxbio. | Michelle Howard-Sparks: Commercial Relationship(s);Code E (Employment):EyePoint Pharmaceuticals, Inc. | Stephan Saint-Juste: Commercial Relationship(s);Code E (Employment):EyePoint Pharmaceuticals, Inc. | Said Saim: Commercial Relationship(s);Code E (Employment):EyePoint Pharmaceuticals, Inc.

Study Group: (none)

ABSTRACT

TITLE: Mechanism of action of vorolanib and differentiation from other anti-vascular endothelial growth factor receptor tyrosine kinase inhibitors

ABSTRACT BODY:

Purpose: Pathological angiogenesis is a hallmark of cancer as well as numerous ocular conditions, including wet age-related macular degeneration (wAMD). As receptor tyrosine kinases (RTKs) such as vascular endothelial growth factor receptors (VEGFRs) play a role in pathological angiogenesis, pan-VEGFR tyrosine kinase inhibitors (TKIs) such as vorolanib, sunitinib, and axitinib are being explored for treatment of these diseases. This present study utilized established methods to compare the antiangiogenic potential of these 3 anti-VEGFR TKIs as well as to differentiate these TKIs.

Methods: Kinase activity was measured using a HotSpot™ assay to identify the TKIs that inhibited RTKs implicated in pathological angiogenesis. For each TKI, half maximal inhibitory concentration (IC_{50}) values for VEGFRs were determined. A human umbilical vein endothelial cell sprouting assay was used to measure in vitro inhibition of angiogenesis, and the chorioallantoic membrane assay utilized to assess in vivo angiogenesis. Computer modeling was performed to examine interactions between vorolanib and VEGFRs because the mode of binding for vorolanib had not previously been characterized. A melanin binding assay was performed for each of the 3 TKIs as an additional method of differentiation.

Results: All 3 TKIs showed strong pan-VEGFR inhibition. In vitro, they effectively inhibited angiogenesis, and in vivo, they were more effective at inhibiting vascular endothelial growth factor (VEGF)-induced angiogenesis than bevacizumab, an anti-VEGF antibody. Based on computer modeling, vorolanib is predicted to be a type II inhibitor of VEGFRs, and this is important as type II inhibitors have greater selectivity than type I TKIs. The TKIs were further differentiated as only axitinib potently inhibited TIE2 (up to 89%) while only sunitinib bound melanin.

Conclusions: Vorolanib, sunitinib, and axitinib bound RTKs implicated in pathological angiogenesis and

exhibited pan-VEGFR inhibition. Only axitinib potently inhibited TIE2, and only sunitinib bound melanin. Retaining TIE2 function is essential for maintaining vascular stability, and melanin binding risks impacting normal cell function. The properties of vorolanib make it of therapeutic interest for treatment of wAMD and other ocular conditions characterized by pathological angiogenesis.

(No Image Selected)

Layman Abstract (optional): Provide a 50-200 word description of your work that non-scientists can understand. Describe the big picture and the implications of your findings, not the study itself and the associated details.: Many eye diseases are associated with changes in the way new blood vessels form. Wet age-related macular degeneration (AMD) and diabetic retinopathy are two examples of eye diseases that have abnormal growth of new blood vessels. A group of medicines called tyrosine kinase inhibitors, or TKIs for short, block the growth of these abnormal blood vessels. In this study, a new TKI called vorolanib was compared with two other TKIs, sunitinib and axitinib. The goal of the study was to see how these medicines were able to block the growth of abnormal blood vessels. It was found that all three medicines worked in a similar way to inhibit this undesired growth of blood vessels. Additional studies revealed that these medicines also have different attributes that differentiate them from each other. The results of this study support doing more studies in the future to see if vorolanib could be used to treat eye diseases such as wet AMD and diabetic retinopathy.

DETAILS

PRESENTATION TYPE - PLEASE NOTE, IF YOU CHANGE YOUR PRESENTATION TYPE AFTER APPLYING FOR AN AWARD (BELOW), YOU MUST GO BACK AND RESELECT THE APPLY BUTTON.: #1 Paper, #2 Poster

CURRENT REVIEWING CODE: 1360 AMD: New drugs, delivery systems, and mechanisms of action - PH

CURRENT SECTION: Physiology/Pharmacology

Clinical Trial Registration (Abstract): No

Other Registry Site (Abstract): (none)

Registration Number (Abstract): (none)

Date Trial was Registered (MM/DD/YYYY) (Abstract): (none)

Date Trial Began (MM/DD/YYYY) (Abstract): (none)

Grant Support (Abstract): No

Support Detail (Abstract): None

TRAVEL GRANTS and AWARDS APPLICATIONS

AWARDS:

© Clarivate Analytics | © ScholarOne, Inc., 2023. All Rights Reserved.
ScholarOne Abstracts and ScholarOne are registered trademarks of ScholarOne, Inc.
ScholarOne Abstracts Patents #7,257,767 and #7,263,655.

@Clarivate | System Requirements | Privacy Statement | Terms of Use

Product version number 4.17.4 (Build 212). Build date Wed Dec 6 07:11:04 EST 2023. Server ip-10-236-29-148
